# Parasite infestations and infections of non-traditional pets and wild mammals: diagnosis and treatment

**DOI:** 10.1590/S1984-29612024074

**Published:** 2024-12-13

**Authors:** Raphael Vieira Ramos, Tiago Manuel Fernandes Mendes, Estevam Lux Hoppe, Darci Moraes Barros-Battesti, Marlene Tiduko Ueta, Silmara Marques Allegretti

**Affiliations:** 1 Departamento de Biologia Animal, Instituto de Biologia, Universidade Estadual de Campinas – Unicamp, Campinas, SP, Brasil; 2 Departamento de Patologia, Reprodução e Saúde Única, Faculdade de Ciências Agrícolas e Veterinárias, Universidade Estadual de São Paulo – UNESP, Jaboticabal, SP, Brasil

**Keywords:** Unconventional pets, ectoparasites, helminths, Pets não convencionais, ectoparasitas, helmintos

## Abstract

Little is known about parasites in wild mammals kept as pets. For this study, fecal and skin/fur samples and ectoparasites from 55 wild and pet mammals attended at a veterinary clinic were evaluated. Opossums (*Didelphis albiventris* and *Didelphis aurita*) were parasitized by helminths (*Aspidodera* sp., *Cruzia tentaculata*, *Trichuris* sp., *Turgida turgida* and Acanthocephala gen. sp.), screw-worm fly larvae (*Cochliomyia hominivorax*), ticks (*Amblyomma dubitatum* and *Amblyomma sculptum*) and fleas (*Ctenocephalides felis*). Hedgehogs (*Atelerix albiventris*) were parasitized by mites (*Caparinia tripilis*), capybaras (*Hydrochoerus hydrochaeris*) by ticks (*A. dubitatum* and *A. sculptum*), a ferret (*Mustela putorius furo*) by fleas (*C. felis*), an orange-spined hairy dwarf porcupine (*Sphiggurus villosus*) by screw-worm fly larvae (*C. hominivorax*) and another for ticks (*Amblyomma longirostre*). Overall, mites were found only in pet animals and helminths were found only in wild animals. Infestation by *Caparinia tripilis* was only found in animals with concomitant illness or stress. *Cruzia tentaculata* actively exited its opossum host by passing through the animal’s anus as its clinical condition worsened.

## Resumo

Pouco se sabe sobre parasitas de animais silvestre mantidos como *pets.* Neste trabalho, foram coletadas amostras fecais, amostras de pelo/pele e ectoparasitas de 55 animais mantidos como *pets* ou de vida livre. Gambás (*Didelphis albiventris* e *Didelphis aurita*) estavam parasitados por helmintos (*Aspidodera* sp., *Cruzia tentaculata*, *Trichuris* sp., *Turgida turgida* e Acanthocephala gen. sp.), larvas de mosca (*Cochliomyia hominivorax*), carrapatos (*Amblyomma dubitatum* e *Amblyomma sculptum*) e pulgas (*Ctenocephalides felis*). Ouriços (*Atelerix albiventris*) estavam parasitados por ácaros (*Caparinia tripilis*); capivaras (*Hydrochoerus hydrochaeris*) por carrapatos (*A. dubitatum* e *A. sculptum*); um furão (*Mustela putorius furo*) por pulgas (*C. felis*); um porco espinho (*Sphiggurus villosus*) por larvas de mosca (*C. hominivorax*) e outro por carrapatos (*Amblyomma longirostre*). O parasitismo por ácaros ocorreu apenas em animais mantidos como *pets*, enquanto os helmintos foram encontrados apenas em animais de vida livre. A infestação causada por *Caparinia tripilis* foi apenas encontrada em animais com doença concomitante ou em situação de estresse. Espécimes de *Cruzia tentaculata* saíram ativamente pelo ânus de um gambá hospedeiro, quando a sua situação clínica piorou.

## Introduction

Mammals are hosts and/or reservoirs of a wide variety of parasites (Santos et al., 2015), Some of these parasites are involved in processes of populational regulation (Tompkins & Begon, 1999; Albon et al., 2002) and ecological community structuring (Thomas et al., 2000), and in many biological processes, such as sexual selection (Ehman & Scott, 2002) and host performance (Albon et al., 2002), predatory and competition dynamics (Prenter et al., 2004) and biological invasion (Tourchin et al., 2002). Parasites are rarely directly responsible for animal death, considering that hosts can have a high parasite load without manifesting clinical signs (Camejo & Loughlin, 2015; Wobeser, 2009). However, parasitism can have a negative effect on the host's health, mainly due to resource competition and energy loss to immune response and tissue reparation, thereby reducing the host's survival or reproductive ability (Wobeser, 2009).

Complex co-evolution and adaptation are essential for a balanced host-parasite relationship (Mitchell, 1991; Ebert & Fields, 2020). There is an epidemiological triad that depends on components of the host-parasite system: the infectious agent, the host and the environment. Furthermore, biotic and abiotic factors can strongly affect disease dynamics, thus favoring or limiting parasites (Turner et al., 2021), including imbalances caused by the domestic or commercial (legal and illegal) environments.

Illegal trading of wild animals is the third most profitable activity worldwide, second only to drug and arms smuggling (Rataj et al., 2011). Many of these animals end up in the market for unconventional pets, which has been gaining space within society over the years (Quesenberry et al., 2020). Some of them are exotic species that have invasive potential and may have high impact on local biodiversity (Rosa et al., 2017). Hence, there is clearly a need to gain knowledge of the parasitic fauna of these mammals and of their role in these mammals’ health, especially with regard to those kept as pets.

## Material and Methods

### Sampling

Specimens of arthropods and helminths, as well as samples of feces, hair and skin were collected from wild and exotic pet mammals attended in a veterinary clinic located in Jundiai, state of São Paulo, Brazil, from January 2017 to December 2019. The individuals were identified by the letter “S” followed by a number.

Feces were collected from animals that presented gastrointestinal disorders or that were attended for the first time and had not received antiparasitic drugs within the preceding six months. Fur samples for trichogram and/or skin scrapings were collected from animals that presented tegument-related clinical signs or clinical suspicion, or from those on which parasites could be seen during physical examination. Macroscopic ectoparasites (ticks, fleas, lice and some mites) were removed with tweezers, placed in hot water (60ºC) and stored in 70% ethanol. Necropsy was performed (with owner permission) on animals that died at the clinic or that were received dead: eight opossums (one *Didelphis albiventris* and seven *D. aurita*) and one capybara (*Hydrochoerus hydrochaeris*).

### Processing

Fecal samples were analyzed using the methods of Willis (Willis, 1921), Hoffmann, Pons and Janer (Hoffman et al., 1934) and direct examination. Egg and parasite morphometry was performed to identify family, genus or species (as possible) (Vicente et al., 1997).

Helminths were cleaned in saline solution and stored in 70% ethanol. For identification, they were diaphanized in glycerin ethanol and evaluated under a bright-field microscope (Leica Microsystems). Helminths and eggs were identified using specific taxonomic keys (Adnet et al., 2009; Matey et al., 2001; Vicente et al., 1997).

Skin and fur samples were placed on glass slides together with saline solution and were observed under a microscope. Ticks were identified using a stereoscope. All parasites were identified using specific taxonomic keys (Aragão & Fonseca, 1961; Linardi & Guimarães, 2000; Onofrio et al., 2006; Villalobos et al., 2016; Iacob & Iftinca, 2018).

### Clinical signs

For clinical evaluation, criteria for apathy, physical pain, pruritus, crusting, skin peeling and other illnesses were established. Individuals that did not display normal species behavior and were lethargic were considered apathetic. Physical pain was considered present in cases of extensive wounds or discomfort during the physical examination, or if the animal presented clinical signs of physical discomfort, in accordance with the criteria for each species (Cubas et al., 2007; Gebhart et al., 2009). Pruritus was considered present when the animals scratched themselves several times during the day and showed discomfort. Crusting was defined by the presence of hyperkeratosis or lesions that generated a tegument of crusty appearance that remained adhered to the skin, while loose epithelium fragments (dandruff) were considered to comprise skin peeling. Animals were considered to be under stress based on behavioral observation of each individual (Cubas et al., 2007) or in situations of recent environmental changes (less than two weeks ago), recent presence of new captivity partners or changes in behavior reported by the owner. Concomitant illnesses unrelated to parasitic diseases were classified as other illnesses.

## Results and Discussion

Biological samples from 55 mammals were evaluated: 23 black-eared opossums (*Didelphis aurita*), 15 white-eared opossums (*Didelphis albiventris*), 8 white-bellied hedgehogs (*Atelerix albiventris*), 4 ferrets (*Mustela putorius furo*), 3 capybaras (*Hydrochoerus hydrochaeris*) and 2 orange-spined hairy dwarf porcupines (*Sphiggurus villosus*).

Among these mammals, 38 individuals (69%) were wild, while 17 were kept as pets (31%). Parasites were found in 42% (23/55) of the cases, with 18 in wild animals and 5 in pets. Ectoparasites were the most prevalent group of parasites (27%), followed by helminths (18%). The ectoparasites found were ticks (9/15), mites (3/15), fleas (3/15), and dipteran larvae (2/15). The helminths found were nematodes (9/10) and acanthocephalans (2/10). The parasites and host species are demonstrated in Table 1.

**Table 1:** Ectoparasites, helminths and their hosts.

**Host**	**Ectoparasites**	**Helminths**
*Atelerix albiventris* (n = 8) y	*Caparinia tripilis* (3/8) _C_	-
*Didelphis albiventris* (n=15) y	*Cochliomyia hominivorax* (1/15) _W_	*Cruzia tentaculata* (1/15)_Wx_ *Trichuris* sp*.* (1/15) _Wx_
*Didelphis aurita* (n= 23) y	*Amblyomma dubitatum* (2/23) _W_ *Amblyomma sculptum* (2/23) _W_ *Ctenocephalides felis* (2/23) _C, W_	Acanthocephala (2/23) _Wx_ *Aspidodera* sp.x (4/23) _W_ *Cruzia tentaculata* (6/23) _Wx_ *Trichuris* sp (3/23) _W_ *Turgida turgida* (1/23) _Wx_
*Hydrochoerus hydrochaeris* (n= 3)y	*Amblyomma dubitatum* (3/3) _W_ *Amblyomma sculptum* (1/3) _W_	
*Mustela putorius furo* (n= 4)y	*Ctenocephalides felis* (1/4) _C_	-
*Sphiggurus villosus* (n= 2)y	*Amblyomma longirostre* (1/2) _W_ *Cochliomyia hominivorax* (1/2) _W_	

c – captivity, w – wildlife, n – number of animals, (x/y) - x: parasitized animals, y: total number of animals.

Mites were only found in animals kept as pets, and ticks were only found in wild animals. Fleas were found in both wild and pet animals, while helminths were only found in wild opossums (*D. aurita* and *D. albiventris*).

*Ctenocephalides felis* was found parasitizing ferrets and two opossums (one kept as a pet and the other, wild). No parasites were found in young opossums (< 7 months). Helminths and ticks were only found in adult opossums. Among helminths, *Cruzia tentaculata* was the most prevalent (7/10).

### White-eared opossums (*Didelphis albiventris*)

**Figure 1:**
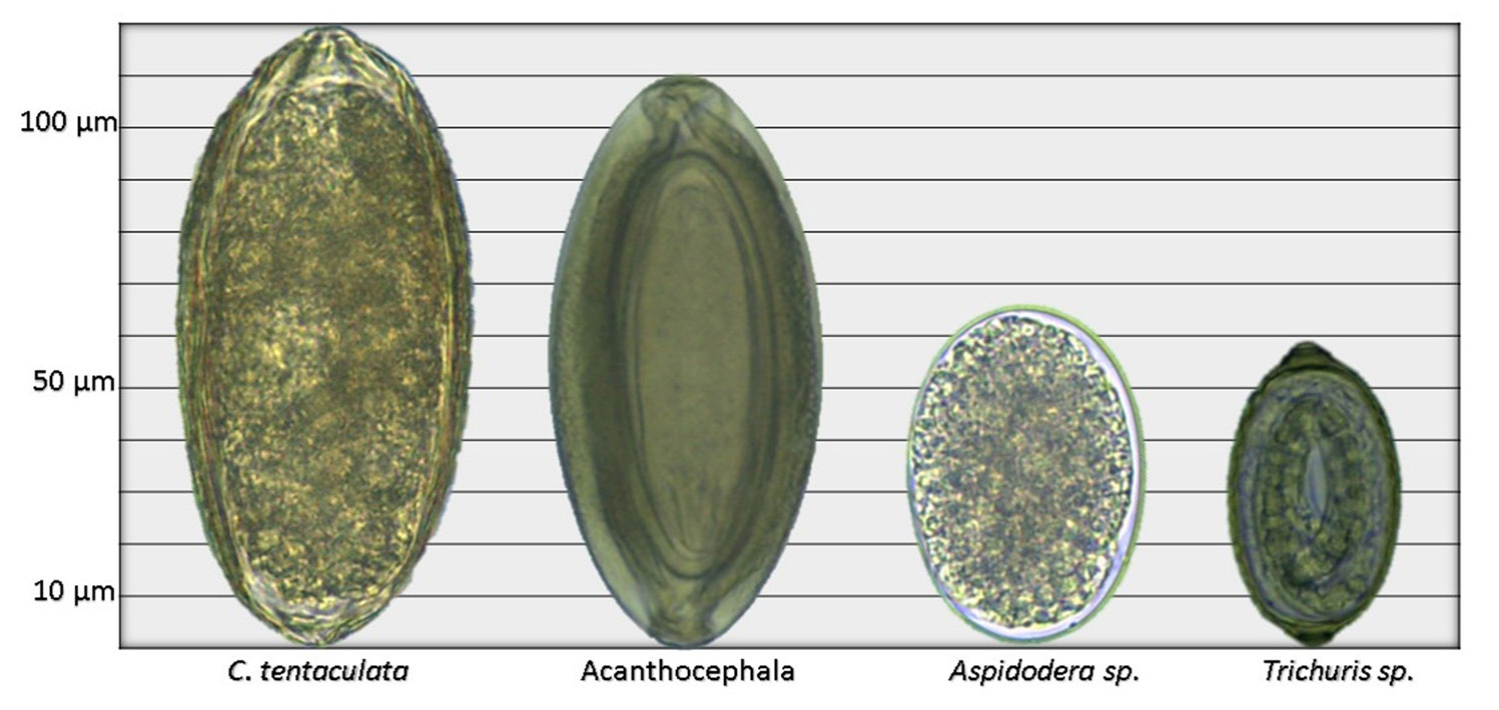
Helminth eggs found in *Didelphis aurita* and *D. albiventris* fecal samples.

**Figure 2:**
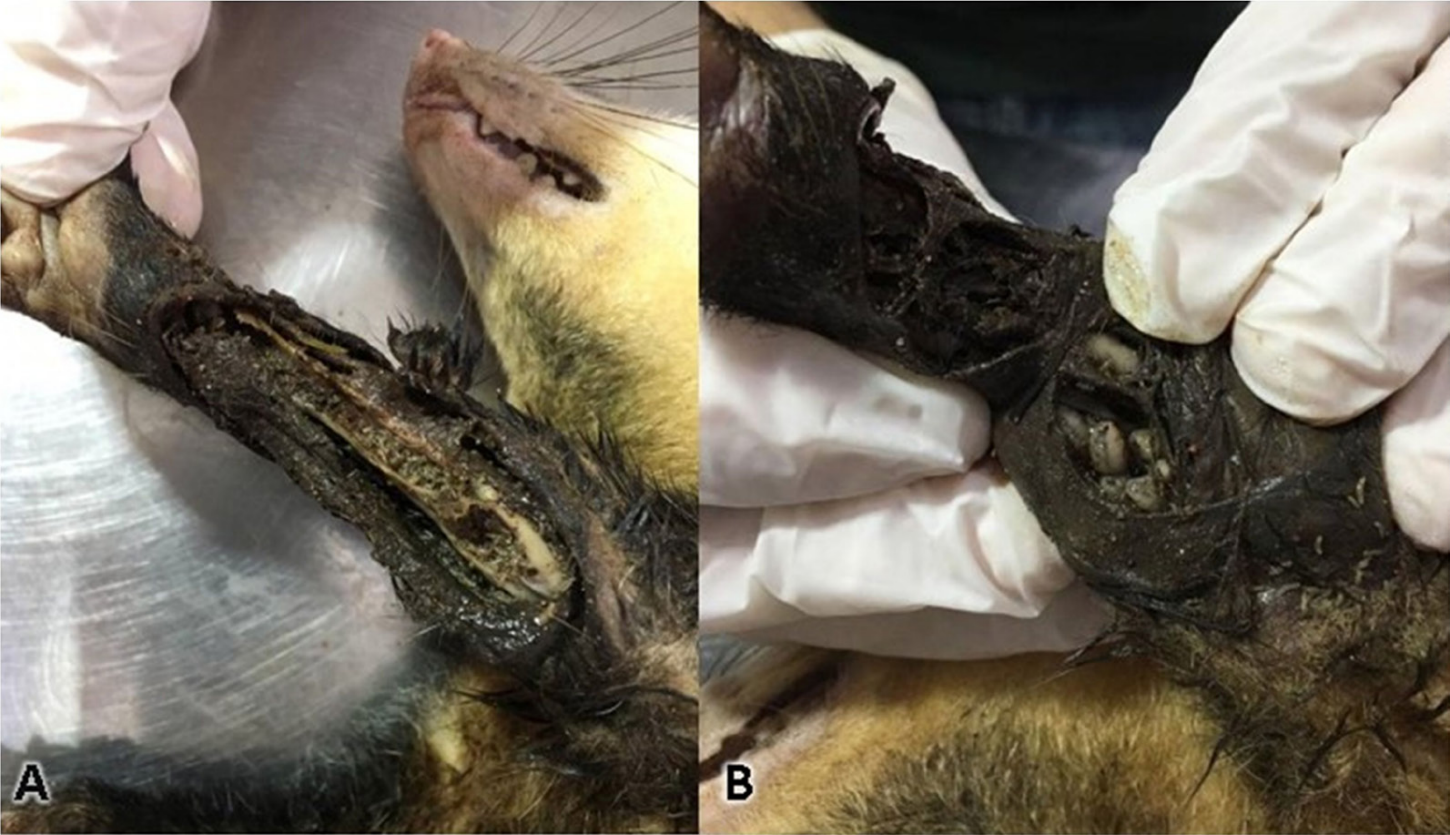
(A) Lesions caused by *Cochliomyia hominivorax* in *Didelphis albiventris*; (B) several *C. hominivorax* larvae in lesion.

Parasites were only found in 2 (2/15) *Didelphis albiventris* (both wild animals). In one individual, *Trichuris* sp. (Nematoda: Trichuridae) eggs (Figure 1) were found in feces**.** In the other individual both *Cochliomyia hominivorax* (Diptera: Calliphoridae) and *Cruzia tentaculata* (Nematoda: Kathlaniidae) were found. *Cruzia tentaculata* eggs were found in feces from the individual S36 and adult nematodes were found in its cecum during necropsy. Recently, it was reported that *C. tentaculata* uses the gastropod *Achatina fulica* as an intermediate host (Ramos-de-Souza et al., 2021). The individual S36 still had eight cubs breastfeeding when found, and fecal examination was performed for 30 days once a week. No parasites were found, thus indicating that transplacental or transmammary transmission does not occur. Myiasis caused by *Lucilia eximia* was reported by Cansi & Bonorino (2011) but, to our knowledge, this is the first report of *C. hominivorax* parasitizing *D. albiventris*. Several larvae in different developmental stages were found feeding on the skin, muscle tissue and bones (radius and ulna) of the carcass of the individual S36 (Figure 2).

### Black-eared opossums (*D. aurita*)

Some individuals were rescued while they were cubs and ended up being kept in captivity because of factors that made release to the wild non-viable (amputations, paraplegia, imprinting etc.). The most common reason why wild individuals of *D. aurita* (both juveniles and adults) were brought for clinical care was dog attacks (9/23; 39.12%).

Parasites were found in 13 (13/23) animals. Only one individual was kept in captivity, alongside cats, and infestation with *Ctenocephalides felis* (Siphonaptera: Pulicidae) was found. *Amblyomma dubitatum* (Acari: Ixodidae) was found in 2 wild animals, one of which, also carried flees (*C. felis*). *Amblyomma sculptum* (Acari: Ixodidae) was found parasitizing 3 individuals.

Helminths were found in 8 (8/23) individuals. *C. tentaculata* is one of the most common opossum parasites in South America (Adnet et al., 2009; Teodoro et al., 2019). Considering only wild animals, overall, 37% (6/11) of *D. aurita* evaluated in this study were positive for this parasite. Among individuals positive for helminths, the parasite was present in 80% (6/8) of the samples.

*Turgida turgida* (Nematoda: Physalopteridae) was found parasitizing the major stomach curvature of individual S58 during necroscopic examination. The location was consistent with the findings of Anderson (2000) regarding parasite location. Different species of the family Physalopteridae are selective towards their location. The result from the coproparasitological examination was negative.

Stool samples from three individuals (S46, S47 and S52) were positive for *Aspidodera* sp. eggs (Figure 1). *Aspidodera raillieti* is the only species that has been reported in *D. aurita* (Costa-Neto et al., 2019).

Acanthocephala eggs were found in a fecal sample from the individual S49 (Figure 1), along with *C. tentaculata* and *Trichuris* sp., but there were no clinical signs of parasitism. An unidentified adult Acanthocephala was eliminated by individual S47 through feces after the animal began to become more agitated and the clinical condition worsened (hypothermia, apathy and intense hemorrhage). The latter individual was also infected with *Aspidodera* sp., *C. tentaculata, Trichuris* sp. and ticks, but did not show any clinical signs related to the parasites. Due to sample deterioration, the adult parasite could not be identified. However, Costa-Neto et al. (2019) found *Oligacanthorhynchus microcephalus* parasitizing *D. aurita,* which, to date, is the only species of Acanthocephala that has been reported in these individuals.

Three fecal samples (S47, S49 and S50) were positive for *Trichuris* sp. eggs. Costa-Neto et al. (2019) reported the presence of *Trichuris didelphis* and *Trichuris diminuta* in *D. aurita*. However, since the eggs of these parasites have similar morphology and sizes, we were unable to identify parasite species through coproparasitological examination. Moreover, no adult nematodes were found.

### *Hedgehog* (*Atelerix albiventris*)

**Figure 3:**
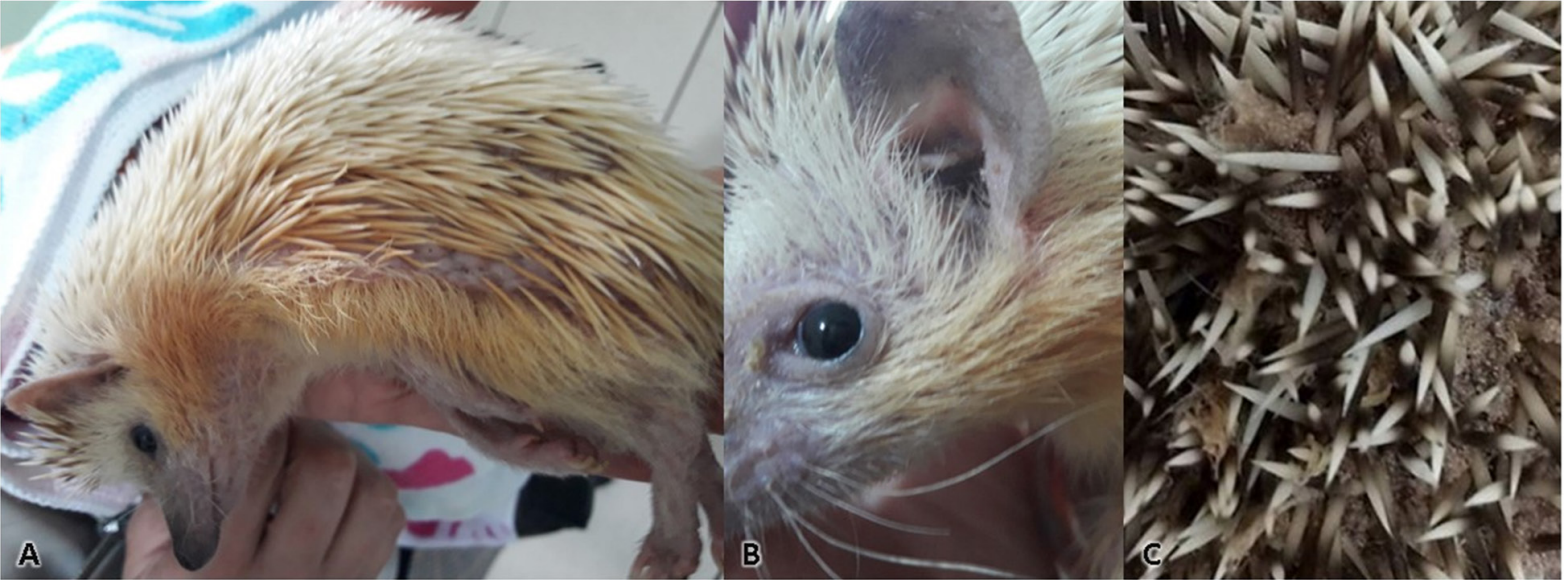
The hedgehog (*Atelerix albiventris*) presents lateral alopecia (A) and crusts on the ear and periocular region of animal S07; (B). Presence of crusts between the spines of animal S02 (C).

*Caparinia tripilis* was found in 3 (3/8) individuals (Figure 3). The clinical signs of their presence include itching, edema, loss of spines, crusted skin and ear lesions and deformation of the base of the nails, and these conditions may result in secondary infections (Mullen & O’Connor, 2002).

One individual presented loss of spines in the left lateral region of its body, along with abundant parasite eggs. After a few weeks, this hedgehog started presenting progressive pelvic limb paresis, with worsening of the parasite infection as the paresis progressed. After treatment for its ectoparasites, which achieved a cure, the paresis progressed to the thoracic limbs. The animal died about two months later, with a postmortem diagnosis of wobbly hedgehog syndrome. In another animal, its apathy improved two days after ovariohysterectomy surgery and the crusting disappeared two weeks after treatment.

Topical treatment with selamectin (6 mg/kg) in a single application proved effective for eliminating parasites and the related clinical signs (crusts, itching and alopecia).

It is worth mentioning that although hedgehogs and *C. tripilis* are both considered non-native in Brazil, one of the hedgehogs with this parasite was collected in the wild. This occurrence serves as a warning about the introduction of non-native species and new parasites, which can significantly impact native species, disrupt ecosystem functions, and ultimately lead to biodiversity loss.

### Capybaras (*Hydrochoerus hydrochaeris*)

Three *H. hydrochaeris* females from a periurban region were attended due to car collisions. During physical examination, they were inspected for ectoparasites. One of the animals was necropsied (S71). Although coproparasitological tests were negative for helminths, all of these animals were parasitized by ticks. *Amblyomma dubitatum* and *A. sculptum* adults were found in S71, *A. dubitatum* nymphs and adults in S69 and *A. dubitatum* adult males in S70. Both of these species have previously been reported in capybaras (Queirogas et al., 2012) and were also found parasitizing *D. aurita* (S47, S55, S57 and S68) from the same region. These tick species were also recorded on free-ranging capybaras in the northeast region of Brazil by Yang et al. (2021) where they found that 71.4% of infested capybaras were positive for rickettsial antibodies while PCR detected rickettsial DNA in 9 out of 9 pools of *A. sculptum* and on 16 out of 19 pools of *A. dubitatum.* This results, along with the presence of free-ranging capybaras in many parts of Brazil, raises serious concerns towards public health.

### Ferret (*Mustela putorius furo*)

One individual (S75) that shared its space with cats was parasitized with *C. felis*. Abundant blackened points were visualized in its coat, and this host presented mild itching, with no apparent lesions. Parasitism is usually asymptomatic in these hosts, but it can cause intense itching and erythematous papules, typically in the dorsal and interscapular cervical region. Moreover, unlike dogs, ferrets hardly ever exhibit hypersensitivity reactions to flea bites (D’Ovidio & Santoro, 2020).

Treatment was performed with a single topical application of selamectin (15 mg/kg), and weekly environmental cleaning consisting of spraying a 0.2% metrifonate solution for a month was implemented.

### Porcupine (*Sphiggurus villosus*)

Only two porcupines were examined during this study. An orfan infant porcupine had, in his dorsal region, an adult male tick of the species *Amblyomma longirostre* which has been previously reported in individuals of the family Erethizontidae (porcupines) (Nava et al., 2010). The other porcupine was an adult found in a private residence backyard. This animal showed apathy, with several lesions throughout the body. *Cochliomyia hominivorax* larvae were found in the cervical region of this individual. To our knowledge, this is the first report of parasitism by *Cochliomyia hominivorax* in porcupines. It was suspected that the animal had been attacked by dogs and that the resulting lesions facilitated myiasis occurrence. The animal died shortly after being rescued.

## Conclusion

In this work, we describe infestations by *C. hominivorax* in *Didelphis albiventris* and *Sphiggurus villosus*, which to the best of our knowledge form the first reports of such infestation*.* The specimen of *D. albiventris* was found dead and presented severe damage to one of its legs. The absence of other lesions or health indicators, along with the presence of live cubs inside this marsupial, seemed to suggest that the damage caused by *C. hominivorax* contributed significantly to the animal death. Regarding the infestation in *S. villosus*, we suspect that this animal was attacked by dogs and that *C. hominivorax* parasitized the lesion, thereby leading to this animal’s death. However, it was impossible to ascertain whether the damage caused by the infestations helped to cause the animal’s death.

The introduction of exotic animals brings the risk of introducing new parasites that might cause severe harm to native species. As mentioned earlier, hedgehogs are not native to Brazil and, therefore, our finding of a wild individual infested with *C. tripilis,* a non-native parasitic mite, raises concerns regarding the potential effect on local species. This also shows one of the problems caused by animal traffic and the introduction of exotic animals through the pet market. Also, the risk of cross-transmission between non-conventional and conventional pets (e.g. cats and dogs) must be kept under watch since the association between animal species that don’t usually associate naturally might facilitate parasites crossing the host species barrier and lead to new diseases that might not behave the same way as when in their natural host.

The clinical manifestations caused by ectoparasites and the elimination of live adult helminths through the host’s anus or in its feces can represent an aggravation of non-parasitic diseases or a sign of stress experienced by the host. Despite the limited number of observations in hedgehogs, mites seem to act as clinical indicators for mammals kept under human care, since ectoparasites' clinical manifestations have been identified as warning signs for other concomitant non-parasitic diseases, which are sometimes asymptomatic. Although more data are still needed, presence of mites might constitute an interesting tool for identifying other pathological conditions in wild animals. Thus, their correlations should be further investigated.
